# 10 years of Malaria Journal: how did Open Access change publication patterns?

**DOI:** 10.1186/1475-2875-9-284

**Published:** 2010-10-14

**Authors:** Marcel Hommel

**Affiliations:** 1 Editor-in-Chief, Malaria Journal, Liverpool, UK

## Background

Fifteen years ago, most publications were paper-based, accessible only by subscription - be it a personal or a library subscription. By the late 1990s, this 'traditional' mode of access to scientific literature was about to change dramatically, as the result of a combination of events: the improvement of personal computer hardware (with computers becoming faster and cheaper every year), public access to internet and the world-wide web, online publishing and, most of all, the will of the scientific community to make research more easily available. This has led to the development of Open Access (OA). This revolution in publishing has given developing countries an opportunity to have access to the latest scientific literature, by-passing the need for libraries, which they could not afford.

Once 'traditional' publishers had started to produce their journals in an online format, it became possible to consider making them available free-of-charge or at low-cost to readers outside the industrialized world: this was first achieved in 2000, through the *HINARI Initiative*, a partnership between the World Health Organization and a number of publishers. It was a step in the right direction, but was far from providing the desired unlimited access.

In 1997, the U.S. National Library of Medicine made *MEDLINE*, the most comprehensive index to medical literature, freely available online in the form of *PubMed*: as a result, the usage of this database increased 100-fold overnight. Since access to research abstracts alone is insufficient, this quickly led to the recognition of the need for an open online repository of full articles, which was realized as *PubMedCentral*. Once this was in place, Vitek Tracz, the chairman of a UK-based publishing company, was able to launch *BioMed Central *in 2000. BioMed Central which is now part of the Springer Group, publishes 206 peer-reviewed Open Access journals, including *Malaria Journal*, started in 2002, and *Parasite & Vectors*, started in 2008. The fact that articles in BioMed Central journals are immediately backed-up in the PubMed Central repository provides them with a long-term security other online journals may not provide.

The Open Access concept really gained momentum when funding agencies in many countries, including the Wellcome Trust, the Department of Health and the Medical Research Council in the UK, made it a requirement in 2006 for the research they had financed to become freely available in open access, not later than six months after its publication. This has forced many 'traditional' non-OA journals to make articles available in limited open access, after an embargo period of 6-12 months or longer. The alternative to publishing in a journal that provides immediate OA to all of its articles on the publisher's website (such as BioMed Central's journals), is for the author to 'self-archive' in a repository (for example, in an institutional repository or in PubMed Central).

The creation of the *Public Library of Science *(PLoS) in 2001, initially as an organization to advocate Open Access publication, led to the creation of a number of PLoS journals initially aimed firmly at the 'high quality end' of the scientific spectrum. The launch of these new journals, together with the wide range of OA journals started a few years earlier by BioMed Central, has changed the scientific publication scene forever.

## How did Open Access affect the publication of malaria research ?

**Figure 1:**
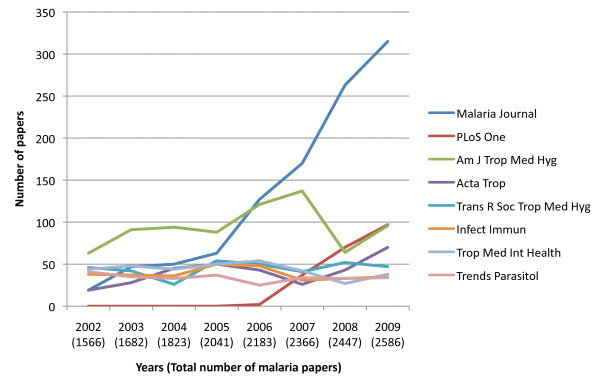
**An analysis of malaria papers publications between 2002 and 2009, based on a PubMed search for 'malaria'**. The total volume of publications per year has been adjusted by -9%, based on the detailed analysis of the 2009 papers. The seven journals publishing the most papers on malaria in 2009, were compared over the 8-year period.

A PubMed search for publications on 'malaria' in 2009, shows 2,839 papers, but as bibliometric tools are imperfect, 9% of these had only a trivial malaria content and were excluded from further analysis; the same correction factor was arbitrarily applied to publications on malaria between 2002 and 2009, as shown on Figure 1. Accordingly, a total of 2,584 papers had been published in English in 2009, in 528 journals (390 of which are impact factor-rated)(see Additional file 1 for the list of journals analysed). The extreme variety of journals publishing papers on malaria is an indication of how wide a field 'malaria' actually covers. While the majority of journals published only one or two papers on malaria that year, the bulk of papers (885, 34.2%) was found in a group of 13 journals, each with more than 25 papers that year. An analysis of seven of these journals over a period of 8 years shows that *Malaria Journal *and *PLos One *now publish 12.1% and 3.7% of all papers, respectively, while the other journals had kept more or less the same volume of publications over the period (i.e. a relative decrease of their share in the total volume of malaria publications, which had increased from 1,566 to 2,584 papers per year over the period). By comparison, in 2002, publication of malaria papers was spread more widely than in 2009, with 30 journals sharing about 25% of the bulk of the publication volume.

The success of the open access movement has led to the launch of many more specialist journals over the past three years, including *PLoS Neglected Tropical Diseases*, *Parasites & Vectors*, *Journal of Infection in Developing Countries*, all in disciplines of interest to the scientific community working on malaria. Furthermore, Hindawi has announced the forthcoming launch of *Malaria Research & Treatment*, whilst the MalariaWorld Newsletter has announced the project of a *MalariaWorld Journal*. In terms of improving the dissemination of information to the widest possible audience, this diversity of journals can only be a good thing. It also provides authors with a choice to best fit their needs.

**Figure 2:**
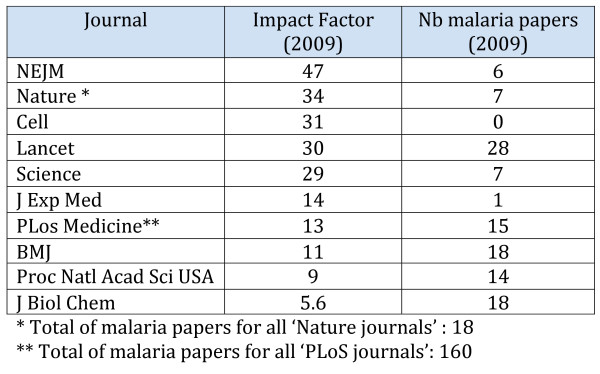
**List of the 'big-10' prestige journals, their impact factors and the number of articles on malaria published in 2009**.

Why do authors choose to publish in one journal rather than another ? An author's ideal choice would deliver fast publication, in a journal that has a broad coverage and a good exposure to colleagues in their discipline, whilst also conveying 'prestige' (often taken as synonymous with having as high an impact factor as possible), and in a journal that is free-of-charge. No journal does, of course, have all these features, but on the whole, open access journals are doing better than traditional journals on most of these although availability of sufficient funding to cover open access publication fees (article processing charges, APC) remains a concern for many authors. It is notable that the 'big 10' - the journals usually considered to convey the most 'prestige' (see Figure 2) - published only 4.4% of the total number of malaria papers in 2009.

Journals available for immediate open access and publishing papers on malaria are still in the minority, but their importance in terms of volume of publication is increasing every year. In 2009, 31 open access journals, including *Malaria Journal *plus 22 other BioMed Central journals, seven PLoS journals and the *BMJ *published 21% of all malaria papers that year. In addition, in view of the global interest in malaria, many non-Open Access journals provide free access to occasional articles on malaria or to special issues on malaria and other tropical/neglected diseases.

## Impact of Open Access on Developing Countries

**Figure 3:**
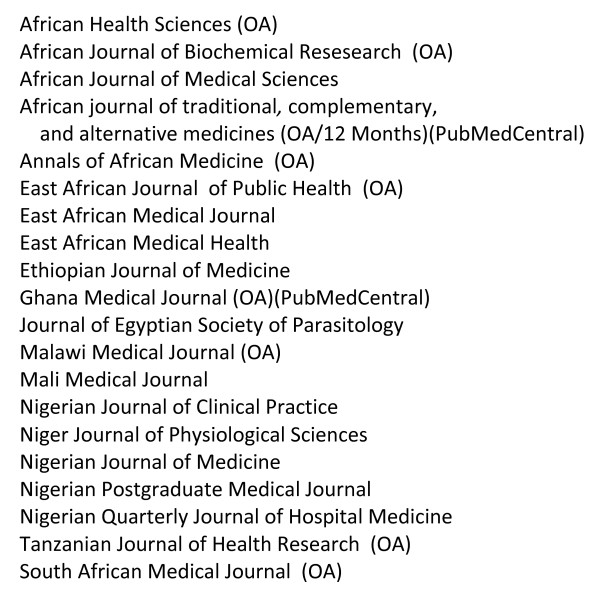
**List of Africa-based journals having published at least one paper on malaria in 2009, showing those that are Open Access and those that use the PubMedCentral repository**. None of these journals are impact factor rated.

Considering that over 90% of malaria mortality occurs in Africa, it is a sad observation that in the 20 Africa-based medical journals listed in the PubMed search for 2009, a total of only 52 articles was published there (see Figure 3). This does not reflect any lack of African medical journals-- there are many -- but rather reflects the fact that many of these are still paper-based, or online via subscription only, are irregular in their production and are often not discoverable via PubMed. There is hope, however, that this situation may improve: hundreds of African online journals are now indexed and archived on AJOL (African Journals Online) and many are moving towards an open access format, sometime with assistance from various Non Governmental Organizations, such as Bioline International. Figure 2 shows that half of the journals listed in AJOL are open access and that two of them are also archived in PubMedCentral (*Ghana Medical Journal *and *African Journal of Traditional, Complementary, and Alternative Medicines*). On the other hand, it is conceivable that the improved access to papers from 'Northern' journals may actually be a disincentive for the redevelopment of Africa-based journals - this would be a shame.

BioMed Central in general, and *Malaria Journal *in particular, are making a special effort to attract articles from authors in the developing world. It is often said of Open Access journals that their APCs are very high and would make it impossible for authors from the developing world to publish in them. In reality, BioMed Central is waiving APCs for authors from the poorest countries (based on a World Bank list), if they request it. In 2009, of the 315 articles published in *Malaria Journal*, 81 had an African scientist as first author and a further 47 had African scientists as one of the authors.

## Malaria Journal

**Figure 4:**
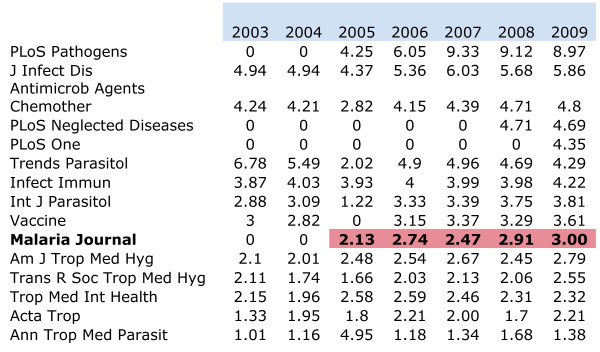
**Evolution of the impact factor of *Malaria Journal *compared to 15 journals, known to publish a substantial number of malaria papers every year**.

The exponential growth of *Malaria Journal *over the past nine years can be explained by a combination of factors: having been the first open access journal in tropical medicine, created at the very start of the Open Access movement, having a prestigious and dynamic Editorial Board, making the effort to copy-edit all articles in a traditional fashion, and helping less-experienced authors from the developing world to reshape their manuscript to an acceptable standard. Figure 4 shows the evolution of the journal over time, in terms of its impact factor, in comparison with others; *Malaria Journal *has now been a leader amongst specialist tropical medicine journals for a few years and the average output this year has reached a paper per day. Quality is not only judged by how often an article is cited, but also by the number of times it is consulted online: in a way, this is how readers show their interest in a paper, and this is recognized on the paper by a 'highly accessed' tag.

**Figure 5:**
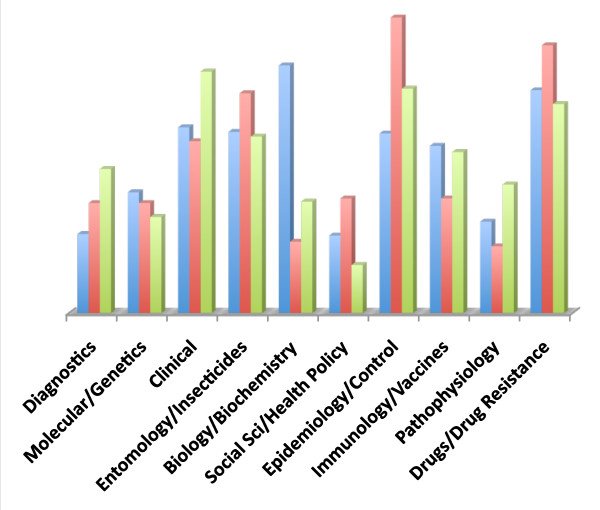
**Comparaison of literature coverage (2009 sample): total papers published on malaria (in blue), *Malaria Journal *papers (in red), *American Journal of Tropical Medicine and Hygiene *(in green)**. The following categories were used: 1. Diagnostics. 2. Molecular/Genetics. 3. Clinical. 4. Entomology/Insecticides. 5. Biology/Biochemistry. 6. Social Sciences/Health Policy. 7. Epidemiology/Control. 8. Immunology/Vaccines. 9. Pathophysiology. 10. Drugs/Drug resistance.

Since its launch almost 10 years ago, *Malaria Journal *has promoted itself as a journal accepting papers on 'malaria in its broadest sense and publishing exclusively papers on malaria'. It was of interest, therefore, to look at its publication output in 2009, compared to the whole field (using the 2,584 papers found through the PubMed search), as well as to one of the most respected 'traditional' tropical medicine journals, the *American Journal of Tropical Medicine and Hygiene *(see Figure 5). While both journals have a broad coverage of the discipline, comparing reasonably well with the overall content of papers published that year, there were substantial differences in the level of coverage: both journals covered the Biology/Biochemistry category poorly, but *Malaria Journal *had a better than average coverage of the Epidemiology/Control, Social Sciences/Health Policy and Entomology/Insecticides categories, while the *American Journal of Tropical Medicine and Hygiene *had a better than average coverage of the Clinical category.

The main thrust of the journal is the publication of peer-reviewed Research papers (87% of papers in 2009), but it also contains Case reports, Methodology papers, Reviews, Opinion and Commentaries. The journal produced a series of supplements, including one on 'The research agenda for global malaria elimination', one on the drug 'Coartem' and one on 'Development of the sterile insect technique for African malaria vectors'; a supplement on 'Natural products for anti-malarial drug development' is in preparation. The journal also offers the possibility of thematic series, which bring together in one section of the journal a number of papers on the same topic; one such series on 'Malaria elimination', guest-edited by M. Tanner, was started early in 2010.

This year, the Malaria Journal and BioMed Central are organizing a three-day Conference on 'Parasite to Prevention', with the abstracts of the presentations and posters presented at the conference to be published in *Malaria Journal*. This is an appropriate way of marking the coming of age of the journal and be able to give some thoughts for its future.

An Editorial to mark to 10^th ^year of *Malaria Journal *could not have been written without acknowledging the work of so many colleagues who have so generously given of their time to peer-review the manuscripts submitted to the journal: their comments have helped to transforms manuscripts into quality papers, and the journal could not have worked without them.

## Supplementary Material

Additional file 1 **List of the 528 journals that have published at least one malaria paper in 2009**. Showing three categories: those have published more than 25 papers, those that have published between 6-24 papers, and those who published 5 or less. Journals that are highlighted in yellow are those that are known to be available for 'immediate' Open Access. Click here for file

