# Supplementary material for: 10 years of Malaria Journal: how did Open Access change publication patterns?
Source: Malar J. 2010 Oct 14;9:284. doi: 10.1186/1475-2875-9-284 (PMC3020680; doi:10.1186/1475-2875-9-284)
Supplement: Additional file 1 — List of the 528 journals that have published at least one malaria paper in 2009. Showing three categories: those have published more than 25 papers, those that have published between 6-24 papers, and those who published 5 or less. Journals that are highlighted in yellow are those that are known to be available for 'immediate' Open Access. [file 1475-2875-9-284-S1.pdf]

JOURNALS WITH MORE THAN 25 ARTICLES

Acta Tropica
American Journal of Tropical Medicine and Hygiene
Antimicrobial Agents and Chemotherapy
Infection and Immunity
Int J Parasitol
Journal of Infectious Diseases
Lancet
Malaria Journal
Plos One
Transactions of The Royal Society of Tropical Medicine and Hygiene
Trends in Parasitology
Tropical Medicine & International Health
Vaccine

JOURNALS WITH 6-24 ARTICLES

Annals of Tropical Medicine and Parasitology
Annals of Tropical Paediatrics
Biochemistry
Bioorganic & Medicinal Chemistry
Bioorganic & Medicinal Chemistry Letters
Blood
BMC Public Health
British Medical Journal*
Bulletin of The World Health Organization
Cell Host & Microbe
Emerging Infectious Diseases
Eukaryotic Cell
Experimental Parasitology
Health Policy and Planning
Indian Journal of Medical Research
Journal of Biological Chemistry
Journal of Ethnopharmacology
Journal of Medicinal Chemistry
Journal of Medical Entomology
Journal of Parasitology
Journal of Travel Medicine
Journal of Tropical Pediatrics
J Vector Borne Dis
The Korean Journal of Parasitology
Lancet Infectious Diseases
Microbes and Infection
Military Medicine
Molecular and Biochemical Parasitology
Nature
New England Journal of Medicine
Parasite Immunology
Parasitology
Parasitology Research
Plos Medicine
Plos Neglected Tropical Diseases
Plos Pathogens
Proceedings of the National Academy of Sciences USA
Science
Southeast Asian Journal of Tropical Medicine and Public Health
Travel Medicine and Infectious Disease
Tropical Doctor

JOURNALS WITH 1-5 ARTICLES

Acs Chemical Biology
Acta Cirurgica Brasileira
Acta Neuropathologica
Acta neurologica Taiwanica
Acta pharmaceutica
Advanced Drug Delivery Reviews
Advances in Experimental Medicine And Biology
Advances in Parasitology
African Health Sciences
African Journal of Biochemical Resesearch
African Journal of Medical Sciences
African Journal Traditional Complement Altern Med
Aids
American Journal of Clinical Nutrition
American Journal of Clinical Pathology
American Journal of Emergency Medicine
American Journal of Epidemiology
American Journal of Health-System Pharmacy
American Journal of Hematology
American Journal of Human Biology
American Journal of Neuroradiology
American Journal of Pathology
American Journal of Perinatology
American Journal of Physiology-Cell Physiology
American Journal of Psychiatry
American Journal of Therapeutics
Amino Acids
Analyst
Analytical and Bioanalytical Chemistry
Analytical Chemistry
Anais da Academia Brasileira de Ciências
Annals of the Academy of Medicine Singapore
Annals of African Medicine
Annals of Biomedical Engineering
Annals of Botany
Annals of Clinical Microbiology and Antimicrobials
Annals of Hematology
Annual Review of Microbiology
Applied and Environtal Microbiology
Archives of Biochemistry And Biophysics
Archives of Gynecology And Obstetrics
Archives of Internal Medicine
Archives of Pharmacal Research
Autoimmunity
Asia-Pacific Journal of Public Health
Aviation Space and Environmental Medicine
Bangladesh Medical Research Council Bulletin
Biochemical and Biophysical Research Communications
Biochemical Genetics
Biochemical Journal
Biochemical Pharmacology
Biochimica et Biophysica Acta-General Subjects

Biochimie
Bioinformatics
Bioinformation
Biologicals
Biology of The Cell
Biomedical Chromatography
Biomolecular NMRAssignments
Bioprocess and Biosystems Engineering
Bioscience Trends
Biosensors & Bioelectronics
Biotechnology Journal
Birth Defects Research Part B-Developmental and Reproductive
BJU International
Blood Cells Molecules and Diseases
BMC Biotechnology
BMC Cell Biology
BMC Ecology
BMC Evolutionary Biology
BMC Genomics
BMC Immunology
BMC Infectious Diseases
BMC International Health and Human Rights
BMC Medical Ethics
BMC Medical Genetics
BMC Medical Research Methodology
BMC Medicine
BMC Microbiology
BMC Molecular Biology
BMC Pediatrics
BMC Pregnancy and Childbirth
BMC Research Notes
BMC Structural Biology
British Journal of Haematology
British Medical Bulletin
Bulletin of Entomological Research
Bulletin of Mathematical Biology
Canadian Medical Association Journal
Canadian Communicable Diseases Reports
Cases Journal
Cell Cycle
Cellular and Molecular Life Sciences
Cellular Microbiology
Cellular Physiology and Biochemistry
Chemical Biology & Drug Design
Chemical Record
Chemical Society Reviews
Chemistry & Biology
Chemmedchem
Chemosphere
Child and Adolescent Psychiatry and Mental Health
Chinese Journal of Integrative Medicine
Chinese Medical Journal
Clinical and Experimental Immunology
Clinical and Vaccine Immunology
Clinical Chemistry And Laboratory Medicine
Clinical Infectious Diseases
Clinical Microbiology And Infection
Clinical Microbiology Reviews
Clinical Pharmacology & Therapeutics
Cochrane Database of Systematic Reviews

Communicable diseases intelligence
Communicative & Integrative Biology
Conflict Health
Contemporary Clinical Trials
Cost Effectiveness and Resource Allocation
Critical Care Medicine
Current Biology
Current Drug Metabolism
Current Drug Targets
Current Genetics
Current Infectious Disease Reports
Current Opinion in Hematology
Current Opinion in Immunology
Current Opinion in Infectious Diseases
Current Opinion in Molecular Therapeutics
Current Opinion in Neurology
Current Opinion in Pediatrics
Current Pharmaceutical Design
Current Problems in Pediatrice and Adolescent Care
Current Topics in Medicinal Chemistry
Current Topics in Microbiology And Immunology
Cytometry Part A
Dalton Transactions
Developmental and Comparative Immunology
Diagnostic Microbiology and Infectious Disease
Drug Metabolism Reviews
Drug Resistance Updates
Eastern Mediterranean Health Journal
East African Journal of Public Health
East African Medical Journal
East African Med Health
Ecohealth
Ecology
Embo Journal
Emergency Medicine Australasia
Environmental Health Perspectives
Environmental Microbiology
Epidemiology and Infection
Ethiopian Journal of Medicine
European Journal of Clinical Microbiology & Infectious Diseases
European Journal of Clinical Nutrition
European Journal of Clinical Pharmacology
European Journal of Drug Metabolism And Pharmacokinetics
European Journal of Human Genetics
European Journal of Immunology
European Journal of Medicinal Chemistry
European Journal of Neuroscience
European Journal of Nutrition
European Journal of Pharmaceutical Sciences
European Journal of Pharmacology
European Journal of Radiology
Evidence-Based Complementary and Alternative Medicine
Evidence-Based Medicine
Evolutionary Applications
Expert Opinion on Pharmacotherapy
Expert Opinion on Therapeutic Targets
Expert Review of Anti-Infective Therapy
Expert Review of Vaccines
Eye
Faseb Journal

Febs Journal
Febs Letters
Fems Immunology and Medical Microbiology
Food And Nutrition Bulletin
Free Radical Biology And Medicine
Fundamental & Clinical Pharmacology
Future Microbiology
Gene
General Hospital Psychiatry
Genes and Immunity
Genetica
Genetics
Genetika
Genome Biology
Genome Medicine
Geospatial Health
Ghana Medical Journal
Global Health Action
Global Public Health
Haematologica-The Hematology Journal
Health Affairs
Health Economics
Health Information & Libraries Journal
Health Policy
Health Research Policy and Systems
Heart And Vessels
Heredity
Human Immunology
Human Molecular Genetics
Human Reproductimmunogenetics
Immunological Investigations
Immunology
Immunology and Cell Biology
Immunology Letters
Immunohematatololgy
Indian Journal of Community Medicine
Indian Journal of Critical Care
Indian Journal of Experimental Biology
Indian Journal of Medical Sciences
Indian Journal of Pathology and Microbiology
Indian Journal of Public Health
Indian Journal of Physiology and Pharmacology
Indian Journal of Pediatrics
Indian Journal of Pharmacology
Indian Pediatrics
Infection Genetics and Evolution
Infectious Disease Clinics of North America
Infectious Diseases Drug Targets
Inorganic Chemistry
Insect Biochemistry and Molecular Biology
Insect Molecular Biology
In Silico Biology
International Immunopharmacology
International Journal of Antimicrobial Agents
International Journal of Biochemistry & Cell Biology
International Journal of Biological Macromolecules
International Journal of Biometeorology
International Journal of Cancer
International Journal of Epidemiology

International Journal for Equity in Health
International Journal of Experimental Pathology
International Journal of Gynecology & Obstetrics
International Journal of General Medicine
International Journal of Health Geographics
International Journal of Hematology
International Journal of Hygiene And Environmental Health
International Journal of Infectious Diseases
International Journal of Pharmaceutics
International Maritime Health
Invertebrate neuroscience
Irish Journal of Medical Science
IUBMB Life
JAMA-Journal of The American Medical Association
Japanese Journal of Infectious Diseases
Japan Journal of Infectious Chemotherapy
Japanese Journal of Veterinary Research
Journal of Alternative and Complementary Medicine
Journal of Antimicrobial Chemotherapy
Journal of Biological Inorganic Chemistry
Journal of Biological Sciences
Journal of Biomedical Informatics
Journal of Biomedical Nanotechnology
Journal of Biomedicine and Biotechnology
Journal of Biomolecular Screening
Journal of Chemical Ecology
Journal of Chemical Information and Modeling
Journal of Child Neurology
Journal of Chromatography A
Journal of Clinical Investigation
Journal of Clinical Microbiology
Journal of Clinical Pathology
Journal of Clinical Pharmacy and Therapeutics
Journal of Clinical Virology
Journal of Controlled Release
Journal of Developmental and Behavioral Pediatrics
Journal of Enzyme Inhibition and Medicinal Chemistry
Journal of Ethnobiology and Ethnomedicine
Journal of Eukaryotic Microbiology
Journal of Gastroenterology and Hepatology
Journal of Gastrointestinal and Liver Diseases
Journal of Genetics
Journal of Health Population And Nutrition
Journal of Histochemistry & Cytochemistry
Journal of Hospital Medicine
Journal of Infection
Journal of Infection and Chemotherapy
Journal of Infection and Public Health
Journal of Infection in Developing Countries
Journal of Insect Physiology
Journal of Insurance Medicine
Journal of Intensive Care Medicine
Journal of Invertebrate Pathology
Journal of Mathematical Biology
Journal of Medical Virology
Journal of Medicinal Food
Journal of Microbiological Methods
Journal of Molecular Biology
Journal of Molecular Medicine-Jmm
Journal of Molecular Modeling

Journal of Natural Products
Journal of Nephrology
Journal of Neurochemistry
Journal of Nutrition
Journal of Obstetrics and Gynaecology
Journal of Ocular Pharmacology And Therapeutics
Journal of Paediatrics And Child Health
Journal of Parasitology Research
Journal of Pediatric Hematology Oncology
Journal of Pharmaceutical and Biomedical Analysis
Journal of Pharmacy and Pharmacology
Journal of Photochemistry and Photobiology B-Biology
Journal of Physical Chemistry B
Journal of Postgraduate Medicine
Journal of Proteome Research
Journal of Proteomics
Journal of Separation Science
Journal of The American Mosquito Control Association
Journal of Theoretical Biology
Journal of Toxicology and Environmental Health-Part A-Current Issues
Journal of Tropical Medicine
Journal of Vector Ecology
Journal of Water And Health
Journal of the Association of Physicians of India
Journal of the College of Physicians and Surgeons of Pakistan
Journal of Egyptian Society of Parasitology
Journal of the Indian Medical Association
Journal of the Pakistan Medical Association
Kathmandu University Medical Journal
Lupus
Malawi Medical Journal
Mali Medical Journal
Marine Drugs
Marine Pollution Bulletin
Mathematical Biosciences and Engineering
Mayo Clinic Proceedings
MCN-The American Journal of Maternal-Child Nursing
Medical Anthropology
Medical and Veterinary Entomology
Medical Hypotheses
Medical Journal of Australia
Medical Letter on Drugs And Therapeutics
Medicinal Chemistry
Memorias do Instituto Oswaldo Cruz
Methods in Enzymology
Methods of Information In Medicine
Minerva Anestesiologica
Mini-Reviews in Medicinal Chemistry
Mitochondrion
MMWR. Morbidity and Mortality Weekly Report
Molecular Biology And Evolution
Molecular Immunology
Molecular Medicine
Molecular Microbiology
Molecules
Mount Sinai Journal of Medicine
Mycoses
National Medical Journal of India
Natural Product Communications
Nature

Nature Biotechnology
Nature Chemical Biology
Nature Genetics
Nature Immunology
Nature Medicine
Nature Methods
Nature Protocols
Nature Reviews Drug Discovery
Nature Reviews Microbiology
Nature Reviews Neurology
Nepal Medical College Journal
Nephrology Dialysis Transplantation
Neuroimmunomodulation
Neurology
Neurotoxicology
New Microbiologica
Nigerian Journal of Clinical Practice
Niger Journal of Physiological Sciences
Nigerian Journal of Medicine
Nigerian Postgraduate Medical Journal
Nigerian Quarterly Journal of Hospital Medicine
Nitric Oxide-Biology and Chemistry
NMR in Biomedicine
Nucleic Acids Research
Open Medicine
Pakistan Journal of Pharmacological Sciences
Parasite
Parasites & Vectors
Parasitology International
Pediatric Emergency Care
Pediatric Infectious Disease Journal
Pediatric Nephrology
Pediatric Research
Peptides
Pflugers Archiv-European Journal Of Physiology
Pharmacogenomics
Pharmacology
Phytochemistry
Phytotherapy Research
Plant Biotechnology Journal
Planta Medica
Plos Biology
Plos Computational Biology
Plos Genetics
Prescrire International
Preventive Veterinary Medicine
Progress in Biophysics & Molecular Biology
Progress in Brain Research
Protein Science
Protist
Protoplasma
Public Health
QJM-An International Journal of Medicine
Rapid Communications in Mass Spectrometry
Resuscitation
Revista do Instituto de Medicina Tropical de São Paulo
Revista Panamericana de Salud Pública
Revista da Sociedade Brasileira de Medicina Tropical
Revista de Saúde Pública

Rheumatology International
RNA-A Publication of The Rna Society
Scandinavian Journal of Infectious Diseases
Science Translational Medicine
Scientific American
Scientific Programming
Seminars in Cancer Biology
Seminars in Immunology
Singapore Medical Journal
Tanzanian Journal of Health Research
South African Medical Journal
Theoretical Population Biology
Thrombosis and Haemostasis
Toxicology and Applied Pharmacology
Toxicon
Traffic
Transfusion
Transfusion Medicine
Transplantation
Trends in Biochemical Sciences
Trends in Biotechnology
Tropical Animal Health and Production
Tropical Biomedicine
Ultrasound in Obstetrics & Gynecology
Ultrastructural Pathology
Vector-Borne and Zoonotic Diseases
Veterinary Parasitology
Virology
Vox Sanguinis
Wiener Klinische Wochenschrift
